# Hope, disappointment and perseverance: Reflections of people with Myalgic encephalomyelitis/Chronic Fatigue Syndrome (ME/CFS) and Multiple Sclerosis participating in biomedical research. A qualitative focus group study

**DOI:** 10.1111/hex.12857

**Published:** 2019-01-10

**Authors:** Eliana M. Lacerda, Clare McDermott, Caroline C. Kingdon, Jack Butterworth, Jacqueline M. Cliff, Luis Nacul

**Affiliations:** ^1^ Clinical Research Department Faculty of Infectious and Tropical Diseases London School of Hygiene and Tropical Medicine London UK; ^2^ Primary Medical Care Faculty of Medicine University of Southampton Southampton UK; ^3^ Department of Immunology and Infection Faculty of Infectious and Tropical Diseases London School of Hygiene and Tropical Medicine London UK

**Keywords:** chronic fatigue syndrome, focus groups, multiple sclerosis, myalgic encephalomyelitis, patient and public involvement, qualitative research

## Abstract

**Background:**

The Clinical Understanding and Research Excellence in ME/CFS group (CureME) at the London School of Hygiene & Tropical Medicine has supported and undertaken studies in immunology, genetics, virology, clinical medicine, epidemiology and disability. It established the UK ME/CFS Biobank (UKMEB), which stores data and samples from three groups: participants with ME/CFS, Multiple Sclerosis (MS) and healthy controls. Patient and public involvement have played a central role from its inception.

**Aim:**

To explore the views of participants with ME/CFS and MS on CureME research findings, dissemination and future biomedical research priorities.

**Method:**

Five ME/CFS and MS focus groups were conducted at two UK sites. Discussions were transcribed and analysed thematically.

**Results:**

A total of 28 UKMEB participants took part: 16 with ME/CFS and 12 with MS. Five themes emerged: (a) Seeking coherence: participants’ reactions to initial research findings; (b) Seeking acceptance: participants explore issues of stigma and validation; (c) Seeking a diagnosis: participants explore issues around diagnosis in their lives; (d) Seeking a better future: participants’ ideas on future research; and (e) Seeking to share understanding: participants’ views on dissemination. Focus groups perceived progress in ME/CFS and MS research in terms of “putting together a jigsaw” of evidence through perseverance and collaboration.

**Conclusion:**

This study provides insight into the emotional, social and practical importance of research to people with MS and ME/CFS, suggesting a range of research topics for the future. Findings should inform biomedical research directions in ME/CFS and MS, adding patients’ voices to a call for a more collaborative research culture.

## 1 INTRODUCTION

The Myalgic Encephalomyelitis/Chronic Fatigue Syndrome (ME/CFS) research group at the London School of Hygiene and Tropical Medicine has been a pioneer in the field of participant‐led research, with patients, carers and charity stakeholders working alongside medical researchers in the planning and development of research projects.^1^, ^2^ The group has developed the UK ME/CFS Biobank^2^ (UKMEB), a biomedical research resource to maximize research efforts, using a similar participatory approach.^1^ Blood samples collected from people with ME/CFS (PWME) are stored alongside those from participants with Multiple Sclerosis and healthy controls, with detailed clinical and socio‐demographic data on each participant.

Our research team (CureME) uses the data and samples from the UKMEB for studies in immunology, genetics, virology, clinical medicine, epidemiology and disability in ME/CFS. We also receive applications from national and international research teams wishing to use biosamples and/or data for studies. Decisions on whether to release samples consider not only the quality of scientific design, but also the potential benefit for PWME.^3^ Within this context, we consider the understanding of the meaning and importance of research findings to PWME of paramount importance, and their resultant views on biomedical research priorities.

ME/CFS is a disease characterized by unexplained incapacitating fatigue for over 6 months accompanied by other variable symptoms, leading to substantial reductions in functional ability;^4^, ^5^ population prevalence rates are estimated at around 0.2% to 0.4%.^6^ At its most severe, it can result in patients becoming housebound, unable to mobilize without support, or sometimes bedridden.^7^ ME/CFS often affects young people and has considerable social and economic impacts, due to its chronic nature.^8^, ^9^ The question of interventions for ME/CFS remains complex and controversial, with differing interpretations of existing research evidence and no universally accepted treatment approach.^10^

Biomedical studies in immunology,^11^, ^12^, ^13^ virology^8^, ^14^ and neurology^15^, ^16^, ^17^ and other specialty fields^18^, ^19^, ^20^ have attempted to explain disease pathways in ME/CFS, but research findings are not always reproducible.^21^ The aetiology remains elusive, and in the absence of any confirmatory diagnostic tests, diagnosis is based on clinical history when other possible causes of fatigue have been excluded. The lack of a diagnostic test or biomarker means that many patients experience scepticism from health professionals, employers and others; this sense of stigma is a substantial emotional burden for many patients.^22^

Multiple sclerosis (MS) was chosen as a comparison disease for ME/CFS, because while MS patients also experience chronic fatigue and disabling symptoms, the aetiology of MS is comparatively well understood with definitive diagnostic tests. People with MS have a broadly similar experience of disability, restricted lifestyle and stress contingent to the illness, but without the specific challenges presented by lack of a diagnostic test or social stigma.

For this study, we explored the ideas of the UKMEB participants with MS and ME/CFS on our preliminary biomedical research findings and their dissemination, and on future biomedical research priorities. The engagement of both groups of study participants was critical to fully explore the different perspectives that might be offered according to their disease narratives.

### 1.1 Aim

To contribute to empowering the voices of the communities involved by exploring the reactions of research participants with ME/CFS and MS to preliminary research findings, their reflections on approaches to dissemination and their views on future biomedical research priorities, building on our established participatory approach in ME/CFS research.

## 2 METHODS

We conducted five focus group interviews in July 2017. Three groups were held in London and two in Norwich. The groups comprised 28 research participants, n = 16 with ME/CFS (nine females and seven males) and n = 12 with MS (seven females and five males).

A moderator led each group supported by a second researcher; no other observers were present. In London, the ME/CFS participants were grouped by gender with the men's group led by a male moderator (LN) and the women's group led by a female moderator (EL). All other groups were mixed gender and led by female researchers (CK and EL). Participants gave written, informed consent prior to taking part. The London and Norwich groups were held on different days with participants attending a presentation of preliminary research findings from CureME prior to their focus group. The programme for the day is shown in Box 1. The questions provided were previously discussed and refined among the authors, with input from an external qualitative researcher.

The research team, together with their qualifications and experience are listed at the end of the paper.

BOX 1 **Program for focus group day**

1

1) Greeting and explanation of the program for day
2) Presentation of research findings in lay terms with time for questions for clarification
3) Informed consent process – explanation (with time for questions) and signatures

1) Lunch interval with lunch provided
2) Focus groups discussions
3) Interval
4) Focus group discussions
5) Conclusions

Group tasks and prompts

Focus group – task 1

1) Tell me what you think about the research findings that were presented?
2) Do the research findings seem to make sense when you think of your personal experience of ME/CFS or MS?
3) Are there any additional questions that you think could be answered by this research?
4) Do you believe the way diagnosis is made may have impacted on the study results, how do you think diagnosis of ME/CFS should be made? (For the groups with ME/CFS only).

Focus group – task 2

During the second part of the discussion, participants were asked to discuss in pairs the following questions.

5) How would you explain the findings to another person who hasn't experienced the disease?
6) How do you think these results should be make public for the research community, the health professionals and the public in general?

After 10 min, each pair were asked to present their ideas. The full group was then asked to discuss the key messages that need to be conveyed and on the best means to communicate them.

### 2.1 Patient and public involvement in study design

The CureME Steering Committee, which includes expert stakeholder advisors, ME/CFS charity representatives, PWME and carers, provided feedback on the design of the study and the program for the focus group days.

### 2.2 Participant selection

Participants were drawn from the 380 people who had contributed data and blood samples to the UKMEB. Initially, 100 potential recruits were randomly selected to include a range of illness severity, illness duration, age and gender with selection thereafter purposive. The research nurse approached participants by telephone and/or e‐mail; 13 individuals who were too unwell to participate, declined or subsequently dropped out.

### 2.3 Venues

The presentations and focus groups in London were held at the London School of Hygiene and Tropical Medicine, and those in Norwich at a conference/event centre. Participants travelling to London were offered reimbursement for overnight accommodation in recognition that some would be too unwell to return home the same day. Both venues were wheelchair friendly, and quiet rooms with sofas were provided for rest breaks.

### 2.4 Content of research presentation

Prior to each focus group, participants viewed a 30‐min presentation (by LN, EL and CK in Norwich; and by LN and JC in London), which included the rationale for carrying out the studies, the recruitment process, and the preliminary study findings. All analyses compared the UKMEB data and samples from PWME, people with MS and healthy participants. ME/CFS participants were subgrouped as mild/moderately and severely affected. The socio‐demographic and symptom progression findings resulted from analyses of a bespoke questionnaire previously piloted by the research group,^3^ while disability and quality of life were measured using the SF‐36v2^TM^ Health Survey.^23^ The laboratory‐based findings included: reports on immune responses (particularly on the number and functioning of natural killer (NK) cells), the presence or absence of antibodies against herpes viruses, and the gene expression profiling of the groups.

### 2.5 Data collection

Focus groups were audio‐recorded and transcribed verbatim. All transcriptions were anonymized using numbered codes for each participant, replacing personal identifiable data with bracketed generic titles. Field notes were taken during the focus groups.

### 2.6 Data analysis

The data analysis was conducted by two qualitative researchers (EL and CM), using thematic analysis to identify “patterns of meaning” across the transcripts of the focus group discussions. This inductive approach followed the procedures for Thematic Analysis recommended by Braun and Clarke.^24^

After listening to the audio‐recordings and reading the transcripts to become familiar with the materials, the researchers (EL and CM) carried out a preliminary analysis on paper to draft initial codes and note issues of interest. The transcripts were then uploaded onto NVivo 11.^25^ This software programme facilitated data management and enabled an audit trail in which decisionmaking processes could be tracked throughout the analysis in a transparent manner. Audit trails provide a recognized strategy for increasing the credibility and reliability of qualitative research.^26^

After data coding and coding‐scheme generation, codes were organized into potential themes to describe the analysed data. In an iterative process, these themes were checked for consistency, coherence, and meaningfulness. To enhance dependability, EL and CM independently analysed all transcripts, holding data review sessions to discuss clustering of codes into themes and emergent theme titles. Negative case analyses (seeking out disconfirming examples) safeguarded against reaching a final thematic scheme prematurely and ensured we represented all views.^27^

## 3 RESULTS

We conducted five focus groups in which 28 participants took part (17 ME/CFS, 11 MS). Table 1 summarizes the composition of the focus groups, and Table 2 summarizes participant characteristics.

**Table 1:** Summary of focus groups

	Location	Members	Group size	Moderator	Duration
Group 1	London	Men with ME	n = 6	LN (male)	1 h 23 min
Group 2	London	Women with ME	n = 5	EL (female)	1 h 12 min
Group 3	London	Women and men with MS	n = 6	CK (female)	1 h
Group 4	Norwich	Women and men with ME	n = 5	EL (female)	1 h 10 min
Group 5	Norwich	Women and men with MS	n = 6	CK (female)	1 h 9 min

**Table 2:** Participant characteristics

Characteristic	ME/CFS participants	MS participants
Gender		
Male	7	5
Female	9	7
Ethnicity		
White (British)	14	8
Indian	1	0
Black British/Jamaican		1
White (nationality other than British)		2
Other ethnicity	1	1
Age group		
≥18 and ≤29	2	
≥30 and ≤39	2	
≥40 and ≤49	4	6
≥50 and <60	8	6
Illness duration		
<2 years	4	1
≥2 years and ≤5 years	4	1
>5 years and ≤10 years	3	3
>10 years and ≤20 years	4	3
>20 years	1	4
Illness severity		
Mild‐moderate	13	‐
Severe	3	‐
Diagnosis		
ME/CFS according to CDC 1994^a^ and Canadian criteria^b^	12	
ME/CFS according to CDC 1994 criteria only^a^	3	
ME/CFS according to Canadian criteria only^b^	1	
MS according to NICE guidelines^c^		12

Diagnostic criteria

a Fukuda K, Straus S, Hickie I, Sharpe M, Dobbins J, Komaroff A. The chronic fatigue syndrome: a comprehensive approach to its definition and study. International Chronic Fatigue Syndrome Study Group. Ann Intern Med. 1994; 953‐9.

b Carruthers B, Jain A, De Meirleir K*,* et al. Myalgic encephalomyelitis/chronic fatigue syndrome: clinical working case definition, diagnostic and treatment protocols. Journal of Chronic Fatigue Syndrome 2003; 115.

c National Institute for Clinical Excellence. Clinical Guideline 8: Multiple sclerosis ‐ Management of multiple sclerosis in primary and secondary care. In: Conditions NCCfC, editor. London: National Institute for Clinical Excellence; 2003.

We present here the five key themes emerging from the thematic analysis, supported by verbatim quotes (Table 3). For ease of reference, quotes are numbered within the text using the label (Q1), (Q2), etc.

**Table 3:** Exemplifying quotes arranged by theme

	Exemplifying quotes
Theme 1: Seeking coherence	Q1. It's a big jigsaw that you need. (P3, male, ME, FG1)
	Q2. I think the reason we don't know what causes CFS is because it's really, really complicated (P16, female, ME, FG3)
	Q3 My understanding was that there seems to be a difference in the way the immune system reacts to the introduction of a pathogen, and this is something to do with the answer we need to find in the future, what's happening to the immune system. (P1, male, ME, FG1)
	Q4. I've always wondered whether some viral infection or something like that was what triggered the MS. (P7, female, MS, FG2)
	Q5. I'm particularly interested in the genetic expression of whether the immune system is reacting properly to pathogens or not. (P4, male with ME, FG1)
	Q6. If they say there's a hereditary link through it, I've got nothing in my family. I'm the first, so what's gone on with my genes to kick this all off? (P24, male, MS, FG5)
	Q7. During that time, in a few weeks leading up to me with months being diagnosed with ME, there were several chemicals used in the house usually by myself, paint strippers to take off all wallpapers, moulds, paints….I just wondered whether there was any connection with chemicals, moulds, on the immune system or that sort of thing? (P19, male, ME, group 4)
	Q8. The fact that Biobank breaks down Canadian criteria and non‐Canadian criteria was important. (P3, male with ME, FG1)
	Q9. There needs to be a lot more grouping. ME is just too wide a term…His is more brain‐based, mine is more, for me it feels more muscle and that sort of side. (P5, male with ME, FG1)
	Q10. There seems to be a fairly big distinction between mild, moderate and severe, and from that last graph it almost looked as if they were two separate conditions because they were so different between the severe section and the mild‐moderate section. (P6, male, ME, FG1)
	Q11. We've got relapsing, remitting, primary progressive, secondary progressive. Is there anything in that? I have no idea, because they obviously present differently and they are treated differently. (P7, male, MS, FG2)
	Q12. We all seem to have had some sort of virus and we all seem to have had some sort of trauma. You put those together with the extraction of the genetic information. Whether there's anything? I don't think you should ignore the virus thing and I don't think you should ignore the trauma side because if you find something out in the genetics, are there triggers that then tie into your psychological and physical findings? (P25, female, MS, FG5)
Theme 2: Seeking a diagnosis	Q13. It's just the hope that you'll know what it is, what the cause is and that there can be a test for it because I think we are treated so differently to any other condition where you can say there's a cause, there's a test for it. (P18, female, ME, group 4)
	Q14. I think that's what we're all desperate to see, isn't it, something that says there's something physically wrong with you’ (P17, female, ME, group 3)
	Q15. I got diagnosed originally with ME. I went through five years with really bad times with it. (P24, male, MS, group 5).
	Q16. Yes, that being left in limbo, you don't know where you are. I was told everything. “You're just depressed. Go and get some more happy pills” and all the rest of this sort of stuff. You know something is wrong, but you're not sure what it is. (P28, male, MS, group 5))
	Q17. It was such a relief to have that labelled and to know that I wasn't going mad in the intervening ten or eleven years or whatever. (P28, male, MS, group 5)
	Q18. I think that was the thing with ME. I couldn't go out and mingle because everyone will just label you a malingerer, “There's nothing wrong. He's got ME. What's that? There's nothing wrong with him.” When you've got MS everyone goes, “Oh.” I've got MS.” “Oh, fine, yes. Alright.” (P24, male MS Group 5)
	Q19. Eventually the diagnosis, I was like, “Oh, thank God for that. Now I can move on.” (P27, male, MS, group 5)
	Q20. Without a clear diagnosis, you don't get the support structure. I was left in limbo, having had one incident, to then survive until I got another incident worthy of note. Then when you've got that they said, “Yes, you've got MS” (P28, male MS, group 5)
	Q21. I think for me, to actually be diagnosed was the biggest kick in the teeth I have ever, ever had. (P23 male, MS, group 5)
Theme 3: Seeking acceptance	Q22. They don't take like an overview, you don't feel held by them, you don't feel safe. Like they're looking at you, I've actually got a GP now that is doing that. (P18, female, ME, group 4)
	Q23. I almost feel like on my record there's so much. I actually found a lump and I delayed for a year going because I was just like, I actually said, “I can't have another thing, like I can't, they'll ban me.” (P18, female, ME group 4)
	Q24. What my neurologist said to me, “Count everything else first, see what else it might be, then the last thing it'll be is MS.” He said, “Don't put it top of the list,” he said, “because you start putting symptoms and ignoring them and thinking it's MS and really there's another big health issue going on.” (P24, male, MS group 5)
	Q25. I learnt an awful lot more about it after suffering and as time goes on you think, “Oh yes, that symptom is compatible,” and understand a lot more. (P19, male, ME, group 4)
	Q26. I'm mentally far stronger now than I was when I was in that two to three year period where work were telling me there was nothing wrong with me and I was only pulling a sickie… Eventually the diagnosis, I was like, “Oh, thank God for that. Now I can move on.” (P27, male, MS, group 5)
	Q27. That was the thing that came up for me as well that I anticipated that people with MS would have probably more fatigue and more restriction socially, but it seemed to be the ME group that were more affected. (P20, female, ME, group 4)
	Q28. When they had the right‐hand chart—and I think it said that socially and mentally we became a bit stronger, if I read the chart correctly—in seeing that I thought, “Well, it's probably because we do have a definite diagnosis of a definite condition that you can definitely get your head round, however long that takes and whatever adaptations you have to make. (P25, female, MS, group 5)
	Q29. You go out on a social event, like a bar you're in or something else, and I still really think harder, “I need a wee, but I don't want to get up because they'll think I'm drunk.” (P24, male, MS, group 5)
Theme 4: Seeking a better future	Q30. My main thing as far as I'm concerned, I think vaccinations are often overlooked. Vaccinations have transformed the health of the world basically, but I do think vaccinations are a trigger for some. (P4, male, ME, group 1)
	Q31. Is there anything being done to say certain viruses could well be the trigger? (P6, male, ME, group 1)
	Q32. During that time, in a few weeks leading up to me with months being diagnosed with ME, there were several chemicals used in the house usually by myself, paint strippers to take off all wallpapers, moulds, paints. (P21, female, ME, group 4)
	Q33. That was the point of doing the gene expression work, is, rather than following up the knowns, and the unknown knowns, etc., is to actually try to look globally at everything we can. There will always be things that we don't even think of to look at, but it is an unbiased approach to try to pull things out. (P8, female, MS, group 2)
	Q34. I'm sat here thinking, “What did I expect out of this?” Maybe somebody's going to say, “Okay, we've found a magical cure,” and obviously what is coming back to me is telling me that I'm not going to get that cure within my lifespan, so possibly a bit sad. (P9, female, MS, group 2)
	Q35. I think most of us realize that probably we're a little bit past the point where we're going to be miraculously cured. Well, I always think that if it happens it's great, but I'm not banking on it happening because then I'm going to be disappointed if it doesn't. (P25, female, MS, group 5)
	Q36. I'm glad I'm participating in this and that, even if I don't get the benefit, the future generations will benefit from it.(P11, female, MS, group 2)
	Q37. It was quite interesting, not positive, I wouldn't say, but interesting to see that the pain with ME, according to your research, is more severe than MS. I found that really bizarre in a ‘We need to study this now,’ kind of way. (P13, female, ME, group 3)
	Q38. An objective measure of that would be to use technology that they have now where you could have the actimeters, like they were showing on the Doctor in the House where they had a watch that measured their activity, their night, their sleep and that sort of thing would then back up actually what has been shown by those questionnaires. Then that would surely be a very good measure of a longitudinal measure of each individual and then compare it with MS. (P14, female, ME, group 4)
Theme 5: Seeking to share understanding	Q39. If it's going out in any form to the general public it wants to be in a readable format, not 500 pages of medical comments that nobody who's going to read it can actually understand. (P6, male, ME, group 1)
	Q40. But you do need to thread a personal story through it somehow, that's the formula, that's what we've always seen, a personal story that illustrates the actual research but it's the kind of story that illustrates. (P1, male, ME, group 1)
	Q41. I think it would be good if you could get the results in a fairly mainstream medical journal. Like, I don't know, the BMJ. You get a synopsis of results or something in the New Scientist publication like that, that reaches quite a wide audience as well. It might get picked up by newspapers and other media. (P19, male, ME, group 4)
	Q42. I think the GP needs to have a one pager of what it is, this is the data behind it, this is the key areas of research. (P16, female, ME, group 3)
	Q43. “Have you read the latest stuff? They've found a cure.” No, they haven't. (P7, male, MS, group 2)
	Q44. It needs to be very to the point and positive, but like you say, not over sensationalized. (P26, male, MS, group 5)
	Q45.I mean you'll be making decisions about how much to let out and when, but in whatever you do the communication needs to be talking about that it is an early stage. This is a journey. (P16, female, ME, group 3)
	Q46. I think one aspect is the timing. I think patients are quite eager to see Results, as soon as possible, because everyone's looking for answers and, obviously, there are key things going on right now, like the NICE review. (P5, male, ME, group 1)
	Q47. There's a long way to go and there's no definite answers yet. (P12, female, MS, group 2)
	Q48. Whatever research comes out, the research team is always very quick to say, they really want to big‐up their own efforts and they'll say, “This is the first evidence that ME is biological.” And it's very frustrating to read that because we know so much other evidence that it's biological and it's not fair to say that's the first. And I think what I'd really like is for researchers to be a lot more collaborative, to talk to others, to build on what others are doing. (P5, male, ME, group 1)
	Q49. We thought it was really impressive that you come out and engaged with us as sufferers and told us where you are and promised to come back and do it again, very impressive, very useful. (P19, male, ME, group 4)
	Q50. It is strange to think or to say, I suppose, that in discussing this, for example, with my boyfriend or my parents, I say, “I really feel honoured to take part in this.” This to me seems like a big deal that each of those little squares on that grid, one of those was me. (P12, female, MS, group 2)
	Q51. We've got our interest or a buy‐in to this process. (P24, male, MS, group 5)
	Q52. We haven't got the magical cure yet, and I don't know how many years it's going to be, but we do have such a fantastic resource that it will enable much more research to be done in ME/CFS, and in MS, by having these samples all collated and so well delineated. (P8, female, MS, group 2)
	Q53. It's whole body stuff. So I'm really glad that that work's happening. I understand it's really hard and it may take quite a while but it's happening, so that's great. (P16, female, ME, group 3)

### 3.1 Theme 1: Seeking coherence: Participants’ reactions to initial research findings

All participants express a compelling need to make sense of their illness experience. Research was seen as a means to achieve this, offering hope of putting together a “jigsaw” of evidence which might provide answers (Q1). Participants seemed to be critically examining the research findings in the light of their own knowledge and personal experience, taking on an “investigator” role, rather than being passive recipients of research information.

Participants suggested that ME/CFS and MS are complex illnesses and that finding answers was also likely to be complex (Q2). They emphasized a need for interconnected thinking in which different physiological factors might contribute to the illness, including immune function (Q3), pathogens, particularly viruses (Q4, Q5), physical or emotional trauma and genetics (Q6). The role of environmental factors, food and chemicals in triggering CFS/ME onset and/or worsening symptoms was of particular interest (Q7).

A major concern voiced by PWME, was the need to differentiate between people whose ME/CFS is defined according to specific diagnostic criteria, and those with other forms of chronic fatigue (Q8), as without such clarification, research results could be distorted or diluted. ME/CFS participants requested that it would be made clear in publications that people with chronic fatigue who do not fulfil the criteria for ME/CFS are differentiated in UKMEB studies.

Participants also noted that subgroups within ME/CFS had differences in onset patterns (eg, viral onset or non‐viral), predominating symptoms (Q9) and levels of severity (ie, mild/moderate or severe). Participants showed particular interest in gene expression results, in which those with severe ME/CFS appeared to be more similar to those with MS than those with mild/moderate ME/CFS (Q10). There was ensuing debate on whether severe ME/CFS might have fundamental differences to mild/moderate ME/CFS, or whether severity in ME/CFS should be regarded as simply a continuous spectrum.

Differentiating different subgroups within MS also emerged as a core interest, with participants citing the categories of “relapsing‐remitting MS,” “primary‐progressive MS” and “secondary‐progressive MS” (Q11). Overall, MS participants felt they had reasonable clarity about illness mechanisms, but wanted to know why MS might be triggered, discussing factors such as genetic susceptibility, viruses and other pathogens and physical or emotional trauma (Q12).

Within an overarching need to find coherence by making sense of often confusing symptoms, one of the most vividly expressed issues was the need for a proven diagnostic test for ME/CFS, which is the focus for the next theme.

### 3.2 Theme 2: Seeking a diagnosis: Participants explore issues around diagnosis in their lives

Participants with ME/CFS described a situation in which lack of a diagnostic test and uncertain aetiology left individuals feeling lost, desperate to make sense of their symptoms, and often unsupported by health professionals (Q13, Q14). Research was depicted not only as offering hope of understanding their symptoms, but also of being able to demonstrate proof of disease to regain self‐respect and respect from others.

In contrast, as MS participants noted, MS is an illness with a known disease process. Nevertheless, during the first years of their illness, three of the MS participants had been mistakenly diagnosed with ME/CFS (Q15), and two had their symptoms attributed to psychological causes. Their accounts of these experiences mirrored those of the ME/CFS participants, describing a sense of “limbo” (Q16), with loss of self‐confidence and feelings of despair. The turning point in their illness trajectory appeared to be receiving a definite diagnosis of MS. One MS participant vividly described his sense of relief at feeling that he was “not going mad,” and to have a label for his illness (Q17). Others described similar feelings of restored coherence, being able to deal with the situation, and being able to communicate their illness to others without fearing judgmental reactions (Q18, Q19). MS participants linked receiving a diagnosis not only with a sense of validation and restored social acceptance, but also the ability to access support (Q20). It is important to emphasize, however, that for some, diagnosis of MS had felt completely devastating, with no positive aspects, described by one as “the biggest kick in the teeth ever” (Q21).

While some participants with MS had experienced diagnosis as pivotal to regaining coherence, validation and self‐respect, participants with ME/CFS were still hoping for a similar turning point. When exploring the meaning of research for their lives, the relationship between physical proof of illness and social acceptance emerged as a central concept, and this will be explored further in the next theme.

### 3.3 Theme 3. Seeking acceptance: Participants explore issues of stigma and validation

For many of the participants with ME/CFS, a central figure from whom they sought acceptance and support was their primary care doctor (GP). In the absence of biomedical tests to prove illness, many of the participants felt that they had struggled to convince their GP that they were physically ill and relationships had become strained. Some participants described experiences of feeling disbelieved, unsupported or not “held” within medical relationships, although others reported that they had a good relationship with their current GP who gave them valued support (Q22).

Illuminating reflections were offered by one participant who had been a GP prior to having ME/CFS. Recalling personal experiences, he described some of the difficulties experienced by GPs when presented with a patient with medically unexplained symptoms. The participant noted how much he had learned as a doctor since experiencing ME/CFS himself (Q25).

Feeling judged as not genuinely ill by employers, benefits agencies and friends or family were also highlighted by ME/CFS participants as well as by MS participants, who recalled similar experiences prior to diagnosis (Q26). ME/CFS and MS participants were intrigued by results from the SF36v2™ questionnaire viewed earlier in the day, suggesting that the impact of illness on social function was worse for participants with ME/CFS than MS (Q27). Both ME/CFS and MS participants hypothesized that this might be due to PWME finding it harder to feel acceptance from others and/or self‐acceptance without biomedical proof of illness (Q28). Yet, it is important to note that participants diagnosed with MS also described situations in which their illness impacted their social confidence. Unpredictable episodes of incontinence or problems with being misjudged as drunk due to poor‐balance could lead to considerable social embarrassment and distress (Q29).

Overall, the impact of illness on social acceptance and confidence emerged as a major concern, which, in addition to the illness symptoms, adversely affected quality of life. Research was widely perceived as offering hope for improved quality of life, and the next theme explores participants’ views on this.

### 3.4 Theme 4. Seeking a better future: Participants’ ideas on future research

Participants’ suggestions on research topics tended, perhaps unsurprisingly, to be illness‐specific.

The ME/CFS participants saw finding a physical cause for the illness as the main priority. They considered that researchers needed to be open to a wide range of possible disease causes including the investigation of immune function, mitochondrial function and potential triggers for ME/CFS such as vaccinations (Q30), viruses (Q31), diet, and chemicals or environmental toxins (Q32). They expressed particular appreciation of the use of gene expression profiling as a means of investigating potential dysfunction across physiological systems (Q33).

Participants also suggested comparing blood samples from PWME diagnosed according to specific diagnostic criteria (eg, Canadian Consensus Criteria) to those with other forms of chronic fatigue, in the hope that this might offer clues about underlying illness mechanisms (Q7). Other comparisons suggested were of onset patterns or predominating symptoms. Participants noted that recovery rates for ME/CFS were better in children than adults, and suggested research to investigate this phenomenon.

In contrast, participants with MS expressed feeling reasonable clarity about the cause of their illness, although the search for a cure felt elusive (Q33). Emotions elicited by taking part in research appeared complex, including pleasure or pride at feeling able to contribute to work which could help future generations, alongside sadness that a cure felt unlikely in participants’ own lifetime (Q34, Q35). Participants were also curious to know why they had developed MS personally, highlighting issues such as heredity, and triggering events such as surgery, injury or other trauma.

Both ME/CFS and MS participants were intrigued by results which had found impact of illness on pain, fatigue and social function to be worse in ME/CFS than MS. Several ME/CFS participants wanted to see such comparisons extended, for example, to compare electronically measured activity and sleep (Q37 Q38).

Dissemination of research results was perceived as a crucial route to influencing attitudes, and this is the subject of our final theme.

### 3.5 Theme 5: Seeking to promote understanding; participants views on dissemination

We asked participants to share their ideas about how research results might best be disseminated, considering target audiences, suggested strategies, hopes and concerns.

Recommended target audiences, predictably, included doctors (especially GPs), PWME or MS and their families or carers, social workers, benefits agency professionals and the general public, especially young people.

Tailoring the style of dissemination to the audience was considered particularly important (Q39), with easy‐to‐read scientific explanations illustrated with personal stories for a general readership thought likely to gain the most media attention, especially if the stories were emotionally compelling or linked to celebrities (Q40). In contrast, for medical and scientific audiences, peer‐reviewed publication in reputable journals was considered essential (Q41), with the addition of brief summaries (“one pagers”) to reach busy GPs or other professionals who might not have time to read longer articles (Q42).

Strategies for publicizing results included mainstream media, such as newspapers, radio and TV. Patient organisations were also thought a valuable conduit for communicating research results. While social media was discussed as a means of communication, this raised more concerns than enthusiasm, due to the challenge of condensing complex topics into relatively few words.

For participants with MS, an overarching priority for dissemination appeared to be updating doctors, people with MS, and their families and friends on research progress without raising unrealistic hopes of cure, which some found distressing or frustrating in the past (Q43). A priority was to ensure that any dissemination was concise, positive and realistic (Q44).

Participants suggested that dissemination of early results, even unspectacular ones, could provide hope, tempered by the realistic acknowledgement that small knowledge increments could gradually lead towards greater understanding (Q47). Participants emphasized the importance of collaboration rather than competition between research teams (Q48).

The concept of research collaboration recurred throughout the discussions, underpinning participants’ hopes for the future. Within this context, they appreciated being treated as respected partners by the research team, listening to their views and keeping them informed (Q49). Participants reflected on their own place within the quest for a better future through scientific understanding, as people who had “bought into” the research process (Q50, Q51) through contributing not only blood samples, but their time and effort, ideas, experiences and hopes; expressing enthusiasm by the possibility that they too could genuinely “make a difference” to the research journey ahead (Q52, Q53).

## 4 DISCUSSION

### 4.1 Summary of key results

In recent years, patient perspectives have become increasingly important in informing the is planning, conduct and dissemination of research.^28^ In this study, participants with ME/CFS and MS illustrated the importance of research as means of seeking coherence to make sense of their illness (Theme 1), seeking diagnostic clarity (Theme 2) and proof of illness by which they could gain acceptance and from the medical profession and from society (Theme 3). Participants offered ideas on future research priorities (Theme 4) and recommendations for dissemination (Theme 5).

A key aim in this study was to elicit patient views on biomedical research priorities for the future. Overall, the research topics proposed fitted already familiar categories, such as immunological and mitochondrial dysfunction in ME, triggers including viruses, toxins and other pathogens, and biomedical differences between different subgroups. For participants with MS, the search for a cure was viewed as paramount, though participants were also interested in investigating genetic, viral, and immunological factors in triggering the illness. While not unexpected, these findings have value in confirming findings from our previous patient consultation work.^2^ What this study adds to our understanding is a vivid contextualization of these specific requests, within a wider and more personal understanding of how biomedical confirmation of an illness, or lack of it, can impact the patient's quality of life, with factors including social acceptance, patient–doctor relationships, self‐confidence and support.

An intriguing finding of the study was the extent to which some of the MS participants reported feeling disbelieved by doctors, employers, or others about their symptoms prior to diagnosis. Their accounts of distress and loss of social confidence resulting from this resonate with the experiences of ME/CFS patients. Within this context, sharing research results was portrayed not only as providing information to medical professionals, families, and others in society, but also as a means of changing attitudes. Participants called for a more collaborative research culture, with greater emphasis on explaining where new knowledge fitted into a wider “jigsaw” of knowledge, and less on apparent “breakthrough” discoveries. Participants expressed pleasure at having played a part in contributing to the current research, tempered with awareness that determination was needed for the research journey ahead.

### 4.2 Strengths and weaknesses

As far as we know, this is the first in‐depth qualitative study to examine the views of both participants with ME/CFS and MS on what biomedical research means to them, on future research priorities, and approaches to sharing and publicizing results.

A strength of using focus groups rather than individual interviews was the extent to which participants were able to interact in their reflections, often eliciting richer and more complex exploration of ideas as the discussions progressed^29^ However, our ability to include individuals who were housebound was limited by using this approach as it required patients to be well enough to travel to a venue for the group. Focus groups may also limit the extent to which individuals shared opinions differing from those held by the rest of the group.^30^

Purposive sampling ensured that the groups included men and women of different ages, illness durations and severities of illness. However, we acknowledge that recruiting participants from individuals already participating in the biomedical research of the UKMEB might lead to higher levels of awareness/comprehension of research than the wider patient population, and that patients who were new/naive to research might have expressed different views.^31^

In this study, all focus group facilitators were from CureME, whose remit is biomedical research. Analysis of transcripts suggested that participants appeared to concur with that approach for ME/CFS research. This is perhaps unsurprising, since participants were recruited from the larger sample of those taking part in the UKMEB. The remit of this study was to elicit participants’ views specifically on biomedical research. Further qualitative research exploring patients’ experiences and views on other aspects of ME/CFS research, particularly interventions for ME/CFS, would help to complete a fuller picture of patient views regarding future research.

This study involved five focus groups. While there is little formal guidance on optimal numbers of focus groups, a study by Guest et al found that 80% of all themes were discoverable within two to three focus groups, and 90% were discoverable within three to six focus groups.^32^ The three ME/CFS groups and two MS groups showed marked similarities on the issues they considered of most importance, enhancing credibility that the findings are an authentic portrait of the study topic. Nevertheless, we do not consider that this study reached data saturation, since additional groups might have elicited fresh views.

In London, the ME/CFS focus groups were separated into male and female groups. With only two gender‐specific focus groups, we are unable to determine whether gender affects views on ME/CFS research. However, analysis of our transcripts suggests that if such differences exist, they are relatively subtle and would need a much larger study to make comparisons.

The study included more ME/CFS than MS participants in a 3:2 ratio. This design was in accordance with the remit of CureME and UKMEB, which were established for accelerating research into ME/CFS. MS participants were made aware from the outset that the research to which they were contributing was primarily aimed at ME/CFS, though with the possibility that results might also help people with MS. We are particularly grateful for their contributions within this context.

### 4.3 How do these findings relate to existing literature?

Placing patient perspectives at the centre of healthcare research has been strongly promoted by many international policy makers,^28^ as well as funders^33^ medical journals^34^ and research institutions.

The UK organization “INVOLVE” was established by the National Institute of Health Research to support active public involvement in medical and social care research (invo.org.uk). According to INVOLVE and others, well‐conducted patient and public involvement (PPI) can improve the relevance of research questions and patient recruitment, leading to results which are more meaningful for patients,^35^ and increasing chances of funding and dissemination.^28^

A growing body of international literature documents patient participation in identifying research priorities in many diseases including HIV/AIDS,^36^ neurological disabilities, rheumatoid arthritis^37^ eczema,^38^kidney disease,^39^ Parkinson's disease^40^, ^41^ and dementia.^42^

Various research approaches have been used to elicit patient views on research priorities including focus groups,^43^, ^44^ PPI consultation workshops^45^ and expert panels,^46^ as well as structured consensus seeking methodologies such as the Delphi technique.^39^, ^46^, ^47^ In this study, the use of focus groups permitted us not only to explore patient views on research priorities, but also to shed light on some of the emotional and experiential reasons behind these priorities, helping to inform our understanding of this complex topic. At the same time, more systematic methodologies, such as the Delphi technique,^46^ could provide more structured conclusions on research priorities than was possible in this study.

In 2017, CureME published a study documenting patient participation in designing the UKMEB.^2^ The current study follows on from that work, illuminating patient reactions to what has been achieved, as well as signposting directions for the future, enabling, participants to become active partners with researchers, rather than passive “research subjects” or recipients of information.

### 4.4 Implications for clinical practice

The study findings highlight levels of distress for ME/CFS patients experiencing disabling symptoms of a disease for which there is no proven biomedical test, shared by MS patients with delayed diagnosis. Exploring the experiences of two patient groups have shed light on some unexpected parallels, which bring into sharp focus the importance of how patients are cared for when diagnosis is unclear, and the deep distress caused when patients feel not accepted or “held” within the patient–doctor relationship.

Cocksedge and colleagues have reflected on the doctor–patient relationship in complex chronic illness, highlighting the role of GPs in “holding” patients at times of uncertainty and fear, even when diagnosis is unclear and uncertain and/or there is no cure for the illness.^48^ Chew‐Graham and others have explored the dilemmas faced by GPs in making diagnosis without confirmatory tests,^49^, ^50^ and the challenges to a GP's role within a healthcare system in which biomedical diagnosis is often a prerequisite for treatment or support.^51^

Several of the MS participants with a delayed diagnosis described how they had felt judged by medical professionals and others as inventing or exaggerating symptoms, an attitude which transformed into supportive acceptance with a confirmed diagnosis of MS. Participants with ME/CFS longed for a research breakthrough to achieve a similar transformation. These mirrored narratives raise the question of whether more progress might be made in treating the patients’ accounts as trustworthy, allowing patients, with or without current diagnosis, to feel validated as people deserving of credence and compassion in the medical relationship.

### 4.5 Recommendations for future research

A key study objective was to find out what patients viewed as important in choosing directions for future research. The findings signpost a diversity of specific topics, including investigating subgroups within ME, immunological and mitochondrial dysfunction the role of chemical and environmental triggers in ME/CFS, and genetic, viral and immunological factors for MS. These research topics are congruent with the intentions of CureME and UKMEB, though the study adds specific suggestions which the team will endeavour to find ways of including within future research collaborations.

Participants called for researchers to be less concerned about claiming a “breakthrough” in medical science for their own work, and to put greater emphasis on contributing to a collaborative integration of research knowledge which might one day lead to a biomedical test, effective treatments or cure. CureME hopes to fulfil that request, not only in future dissemination, but in all interactions with the wider research community. Ours and other similar initiatives appear to indicate that the prevailing research culture is changing gradually towards the collaborative ethos participants wish to see.

This study offers a compelling, patient‐centred argument for a paradigm shift within research culture towards collaboration, not only between different research teams but between patients and researchers. How specifically this might be achieved remains a subject for ongoing discussion and development within the ME/CFS and MS research communities, but the participants expressed hope that their contributions might help build a better future for current and future generations, resonates with the ethos we share with many other researchers.

## 5 CONCLUSION

The findings of this study provide insight into the emotional, social and practical importance of research to MS and ME/CFS patients as well as suggesting a range of specific research topics for the future. Findings should inform the future direction not only of the UKMEB, but also of researchers across ME/CFS and MS research, adding the voices of patients to a call for developing a more collaborative research culture.

## AUTHORS’ CONTRIBUTIONS

The research team comprised: EL—MD, PhD, female, extensive experience in qualitative/quantitative health research, including ethics and biobanking; CM—PhD, female, extensive experience in qualitative/quantitative research;, CK—Research Nurse, MSc, female, extensive experience with health research, ethics, and biobanking;, LN—MD, PhD, male, extensive experience with health research, including epidemiology and clinical trials;, and JC—PhD, immunologist, wide experience in disease pathogenesis research, including studies analysing gene ‐expression profiles.

## CONFLICT OF INTEREST

All authors confirm that they have no financial, personal, politic or academic conflict of interest.

## ETHICAL APPROVAL

This study received approval from the London School of Hygiene and Tropic Medicine on (LSHTM Ethics Ref: 14191, 13.06.2017). The study was conducted outside the UK National Health Service, and therefore, did not require review by an NHS Research Ethics Committee.
